# Patients’ Perceptions of Nusinersen Effects According to Their Responder Status

**DOI:** 10.3390/jcm13123418

**Published:** 2024-06-11

**Authors:** Charlotte Lilien, Eva Vrscaj, Gita Thapaliya, Nicolas Deconinck, Liesbeth De Waele, Tina Duong, Jana Haberlová, Markéta Kumhera, Geertrui Peirens, Lena Szabo, Valentine Tahon, Whitney J. Tang, Noor Benmhammed, Laurie Médard, Laurent Servais

**Affiliations:** 1 MDUK Oxford Neuromuscular Centre, Department of Paediatrics, University of Oxford, Oxford OX3 9DU, UK; charlotte.lilien@paediatrics.ox.ac.uk (C.L.); gthapa02@gmail.com (G.T.); 2 Division of Child Neurology, Reference Center for Neuromuscular Diseases, Department of Paediatrics, University Hospital Liege & University of Liege, 4000 Liege, Belgium; eva.vrscaj@kclj.si (E.V.); noor.benmhammed@citadelle.be (N.B.); laurie.medard@citadelle.be (L.M.); 3 Department for Pediatric Neurology, University Children’s Hospital, University Medical Centre Ljubljana, Bohoričeva 20, 1525 Ljubljana, Slovenia; 4 NMRC UZ Gent, Ghent University Hospital, 9000 Ghent, Belgium; nicolas.deconinck@hubruxelles.be (N.D.); valentine.tahon@uzgent.be (V.T.); 5 Neuromuscular Reference Center and Department of Paediatric Neurology, Hôpital Universitaire des Enfants Reine Fabiola, HUB, Université Libre de Bruxelles, 1020 Brussels, Belgium; 6 Department of Paediatrics, University Hospitals Leuven, 3000 Leuven, Belgium; liesbeth.dewaele@uzleuven.be (L.D.W.); geertrui.peirens@ganspoel.be (G.P.); 7 Department of Development and Regeneration, KU Leuven, 3000 Leuven, Belgium; 8 John W. Day Lab, Department of Neurology and Neurological Sciences, University of Stanford, Stanford, CA 94305, USA; trduong@stanford.edu (T.D.); whitneyt@stanford.edu (W.J.T.); 9 Department of Paediatric Neurology, Second Faculty of Medicine, Charles University and Motol University Hospital, 150 06 Praha, Czech Republic; jana.haberlova@fnmotol.cz (J.H.); marketakumhera@gmail.com (M.K.); 10 Paediatric Center, Semmelweis University, 1083 Budapest, Hungary; szabo.lena@semmelweis.hu

**Keywords:** spinal muscular atrophy, nusinersen, responder, treatment response, patient perception

## Abstract

**Background and Objective:** Patients with spinal muscular atrophy (SMA) treated with a disease-modifying therapy (DMT) are often classified as responders or non-responders based on the attainment of a specific improvement threshold on validated functional scales. This categorization may significantly impact treatment reimbursement in some countries. The aim of this research is to evaluate the perception of treatments and their benefit by patients considered as responders or non-responders. **Methods:** In this non-commercial multicenter study, 99 post-symptomatically treated SMA type I–III patients with a median age of 11.2 (0.39–57.4) years at treatment initiation were stratified into three groups based on their treatment outcomes, i.e., those exhibiting clinically significant improvement (N = 41), those with non-clinically significant improvement (N = 18), or those showing no improvement (N = 40). Fifteen months after treatment, the initiation patients or patients’ caregivers were assessed using a patient-rated scoring system based on the Patient Global Impression of Change (PGIC) scale, comprising 22 questions targeting important aspects and tasks in the daily life of patients with SMA. **Results:** We found no statistical difference in the patient perception of treatment benefits in 17 out of 22 domains across patient groups. **Conclusions:** Our results suggest that functional motor scales do not recapitulate patients’ and patients’ caregivers’ experience of the effect of nusinersen treatment in SMA.

## 1. Introduction

Spinal muscular atrophy (SMA) is a severe, progressive, and life-threatening neuromuscular disorder caused by mutations in the survival motor neuron 1 (*SMN1*) gene, leading to a deficiency in the SMN protein, with an incidence of 1:14.848 births [1,2]. The severity of the phenotype is mostly driven by the low and variable residual levels of the SMN protein produced by the autologous gene *SMN2,* for which human beings comprise zero to several copies [3]. Patients with SMA are classified at diagnosis according to the age at onset of symptoms and their highest motor milestone achieved: SMA type I patients show their first symptoms before 6 months and are never able to sit independently; SMA type II patients present with their first symptoms between 6 and 18 months and are able to sit independently; SMA type III patients experience their first symptoms after 18 months and are able to walk; SMA type IV patients show their first symptoms in adulthood, following the achievement of ordinary motor milestones in childhood [4]. As patients may lose their highest motor ability over time, a functional classification between non-sitters, sitters, and walkers to indicate the current phenotype of the patient is more commonly used [4]. Nusinersen, an antisense oligonucleotide targeting pre-mRNA splicing of the *SMN2* gene, was approved by the United States Food and Drug Administration (FDA) and the European Medical Agency (EMA) in 2016 and 2017, respectively, and is one of the three currently approved DMTs for SMA [5,6]. Large real-world data collections have since then provided additional evidence of efficacy in a broad range of patients [7,8,9,10,11,12,13,14]. The vast majority of these controlled and real-world studies were based on the use of well-validated scales, such as the Children’s Hospital of Philadelphia Infant Test of Neuromuscular Disorders (CHOP-INTEND) and Hammersmith Infant Neurological Examination—Section 2 (HINE-2) in type I SMA, and Hammersmith Functional Motor Scale Expanded (HFMSE), Revised Upper Limb Module (RULM), and Motor Function Measure 32 (MFM32) in type II SMA [13,15]. Although these scales have been demonstrated to constitute robust tools to compare populations, individual meaningfulness, especially in older and weaker patients, is not as well established [16]. This becomes an issue in outcome-based agreements when payers and healthcare systems base their judgment to continue funding treatment at the individual patient level using the magnitude of change in these scales determined by the minimal clinically important differences (MCIDs) as the primary driver in the decision-making process [16,17]. The MCID is usually based on a specific population, typically validated from homogenous and clinical trial-based research groups, leading to greater variability and potentially higher MCIDs for the real-world population [18]. Indeed, it has been shown that patients and caregivers are perceiving improvements that are not captured by the functional motor scales [8,10,19]. Although such small improvements not captured by standardized scales may appear insignificant, they can have a considerable positive impact on a patient’s daily life [20,21]. The main objective of this study is to compare the treatment benefits as perceived by the patients and caregivers and as measured by current functional scales.

## 2. Materials and Methods

Participants and study design: Six centers in four countries participated in the study, as follows: Belgium (Leuven, Gent, and Liège—coordinating Centre), Hungary, the United States of America, and the Czech Republic. Data were centrally monitored by the coordinating site, and queries were sent for missing or aberrant data. The study was non-commercial (academic), multicentric, and prospective. Patients with 15 months of nusinersen treatment who had been evaluated by a standardized assessment were proposed to participate. Ethical approval to conduct the research was granted from relevant bodies within each participating center, as dictated by respective local ethics committees. Informed consent was obtained from all subjects and/or legal caregivers involved in the study.

We included patients with genetically confirmed 5q SMA treated post-symptomatically with at least 12 months of a stable dose of nusinersen. Exclusion criteria were patients identified by newborn screening, pregnancy or breastfeeding, the presence of any comorbidities interfering with functional scale assessments and scores, patients with no baseline visit unless the patient had a score of zero points on the functional scale at month 15, and assuming the patients was assessed with a scale in which a floor effect was achieved. Given the heterogeneity in real-world data collection related to timings and outcome choices and to increase the robustness of the study population, we defined the following based on the literature: (i) clear study windows for treatment initiation and assessments, and (ii) an order of priority of functional scales depending on age at treatment initiation and the type of SMA based on the literature (Table S1). This enabled us to objectively prioritize scales for each phenotype and age group and to manage heterogeneity across each country due to the large population and the different scales used across each country. The scales considered were those used in pivotal or phase 2 clinical trials in type I (CHOP-INTEND, HINE-2), type II and type III (HFMSE, RULM, MFM32), and ambulatory type III (6 min walk test (6MWT)), for which minimally clinically important differences were defined [5,6,13,22,23,24]. To be consistent with the other categories, we prioritized a motor function scale like the HFMSE instead of the 6MWT for the ambulant patients. Furthermore, we also considered the CHOP-INTEND for all non-sitter patients regardless of their age, as it was most widely used [25].

To better understand the individual disease trajectory, events happening in the time frame between the baseline and month 15, such as the implementation of tracheotomy, non-invasive ventilation, or gastrostomy, hospitalization in an intensive care unit, and major surgery (e.g., spinal fusion) were considered as adverse events as these can impact the score changes over time [26].

Furthermore, as contracture assessment is usually not harmonized in real-world data collection, we defined contractures as any presence of contracture regardless of the degree of range of motion.

Once the study population was identified and included, patients were divided into three groups (Non-responder (NR); Responder non-clinically significant (RNCS); Responder clinically significant (RCS)) according to their responsiveness to treatment related to functional scales score thresholds commonly used to distinguish treatment efficacy [5,6,24,27] (Table S2). The rationale for considering three groups is that certain payers may consider that the patients need to improve on these scales, and others may consider a certain threshold to confirm the improvement as clinically relevant.

Assessment: We assessed patients—or caregiving parents in patients younger than 12 years—with a questionnaire composed of 22 questions targeting important aspects and tasks in the daily life of patients with SMA. This questionnaire was designed by the authors based on experience in treating and following patients with SMA (Table S3). The objective was not to validate a new questionnaire but to use a well-established method like the Patient Global Impression of Change (PGIC) to measure the perception of change for each item [10,28]. Question 1/22 was about the general impression of change in patients and caregivers after 15 months of treatment. We first explored the presence of an improvement (“Yes/No”) and then asked to score the perceived change with the PGIC. Except for the first one, questions were composed of two sub-questions as follows: (a) a baseline question: patients or caregivers were asked to reply “Yes/No” as to whether they noticed symptoms or any difficulty with one specific task before treatment initiation; (b) the PGIC question: patients or caregivers were asked to score the potential change perceived compared to before treatment initiation to correlate their condition before treatment initiation with their condition following the first year of treatment, as reflected by daily tasks. The PGIC scale [28] uses 7 ratings scored from very much improved (rating 1), being the same (rating 4), to very much worse (rating 7), allowing the patient or caregiver to estimate whether a change is perceived after a year of stable nusinersen treatment. To avoid any confusion from the patient, we reversed the scoring system, such that lower scores represented worsening (scores 1–3) and higher scores represented the improvement (scores 5–7) of the tested task. Question 22 is related to pain and comprises an additional question about whether pain was limiting the patient’s activity in daily life.

Statistical analysis: Data were analyzed using the Statistical Package for the Social Sciences (SPSS) version 29.0. Variables were tested for normality using a Shapiro–Wilk test. Descriptive data are expressed as the median (minimum–maximum) for non-normally distributed continuous data and as proportions for categorical variables. Differences in demographic characteristics and questionnaire responses across the three groups were examined using the Kruskal–Wallis test for continuous non-normally distributed data and ordinal variables (questionnaire items), whereas chi-square tests were used for categorical variables. Significant results (*p* < 0.05) were followed up with pairwise comparisons with a Bonferroni correction for multiple comparisons.

Initially, we examined the perception of change for each of the 22 questions among all patients across the three patient groups. Subsequently, in the 21 questions in which a baseline question was included, we conducted further analyses by differentiating the number of patients who noticed or did not notice symptoms or any difficulty with one specific task before treatment initiation. Statistical analyses were performed only when each questionnaire item (reporting yes or no) had a minimum of 10 patients represented across at least two patient groups. The PGIC score for each question was used to determine whether patients perceived themselves as worsened (scores 1–3), unchanged (score 4), or improved (scores 5–7) after 15 months of treatment initiation (Figure S1).

## 3. Results

A total of 99 participants were studied (see Table 1). Of these, 41 were classified as RCS, 18 as RNCS, and 40 were NR. The distribution was large, but the comparison between groups did not reveal any statistical difference in sex, *SMN2* copies, ambulatory status, patients across outcome groups, adverse events, scoliosis surgery, or feeding assistance.

Significant differences were observed in the age at treatment initiation between the RCS and NR groups, with patients in the RCS group initiating treatment at a younger age (median age 5.20 years, range 0.39–47.8) compared to those in the NR group (median age 18.1 years, range 0.83–57.4), *p* < 0.001. Additionally, greater physiotherapy provision was observed for the RCS group compared to the NR group, and interestingly, less provision was observed for the RNCS group compared to the NR (*p* < 0.01).

Except for RCS at the neck and right wrist/hand, patients across all groups presented contractures, with statistical differences observed between the right hand/wrist (RNCS > NR, *p* < 0.001) and left hand/wrist (RCS < RNCS, *p* < 0.01).

Ventilatory support and spinal surgery showed statistical differences between the different patient groups, with a higher rate observed in the NR group and a lower rate in the RCS group. However, there were no differences between groups in spinal surgery incidence after nusinersen initiation.

Table 2 presents the difference in the prioritized functional assessment depending on age at treatment initiation after 15 months of nusinersen.

HFMSE was the first in line in our prioritization list (Table S1) in patients older than 2 years (51/99 patients). Using HFMSE, 24 patients were classified as NR with a homogenous distribution between adults (n = 13) and children (n = 11), while RCS (n = 27) comprised 22 children.

In our cohort, CHOP-INTEND only assessed children with an equal distribution across the three patient groups (RCS n = 5; RNCS n = 3; NR n = 3).

Considering the entire population, the score differences between the baseline and month 15 in the RCS group were higher in children than in adults (Table 2).

**Figure 1:**
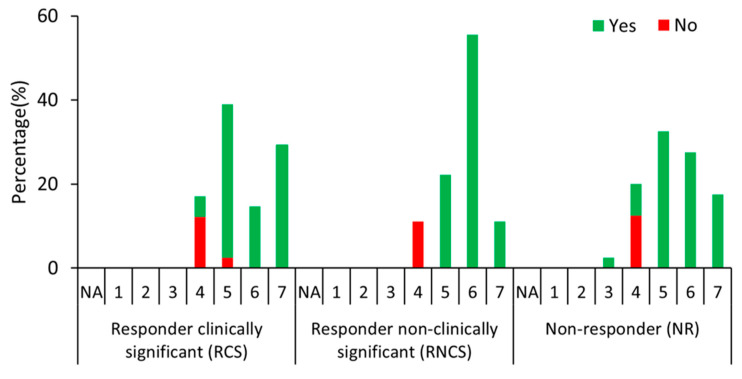
The general impression of change in quality of life. Ninety-nine patients were classified as RCS (n = 41); RNCS (n = 18); and NR (n = 40). The x-axis shows the PGIC score of 1 to 7 (with 1 being much worse, 4 being the same, and 7 being much improved). The number of patients reporting yes/no showing an improvement after 15 months following treatment initiation are in green and red, respectively. The y-axis shows the percentage of participants reporting yes/no to the presence of a symptom or difficulty at the baseline, along with their PGIC scores across the three groups. NA = not applicable. No statistical difference was found across the three patient groups.

No statistical difference between RCS, RNCS, and NR could be found in the general impression of change in quality of life (QoL) (Figure 1; Table S4). More than 78% of patients classified as NR reported an improvement in QoL (PGIC scores >4). On the other hand, approximately 12% of the RCS perceived no change in their QoL.

For the other questions assessing specific fields of potential improvement, we found no statistical difference in 16 out of 21 questions across the three groups (Table S4).

Next, we considered patients who reported difficulties at the baseline for each of these 21 questions (baseline question). Again, we did not find any statistical difference between patient groups in 17 out of 21 questions (see Table 3).

**Figure 2:**
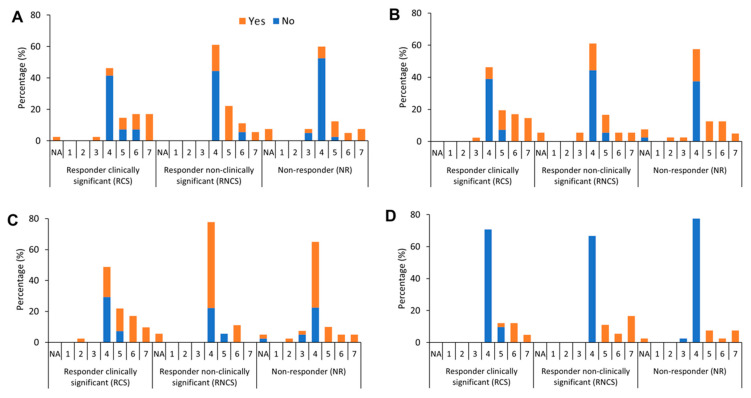
Statistical differences of the PGIC scores across the three patient groups depending on the baseline question. (**A**) Balance while sitting; (**B**) function involving arm muscles; (**C**) function involving shoulder muscles; and (**D**) loudness of voice. Ninety-nine patients were classified as RCS (n = 41); RNCS (n = 18); and NR (n = 40). The x-axis shows a PGIC score of 1 to 7 (with 1 being much worse, 4 being the same, and 7 being much improved). The number of patients reporting yes/no regarding symptoms/difficulty before treatment initiation are in orange and blue, respectively. The y-axis shows the percentage of participants reporting yes/no to the presence of a symptom or difficulty at the baseline, along with their PGIC scores across the three groups. NA = not applicable. Statistical differences across the three patient groups depending on the baseline question were found for these four domains.

Considering the patients who reported no difficulties at the baseline, we did not find any differences between groups in 18 out of 21 questions (see Table 3). The only consistent differences in PGIC for the different groups were changes in balance while sitting (RCS > NR, *p* ^adjusted^ = 0.04) (see Figure 2A), changes in function involving shoulder muscles (RCS > NR, *p* ^adjusted^ = 0.04) (see Figure 2C), and changes in their loudness of voice (RCS > NR, *p* ^adjusted^ = 0.04) (see Figure 2D).

Considering the patients who reported difficulties at the baseline, we found differences in PGIC scores in only two questions amongst the patient groups as follows: change in shoulder muscle function (RCS > RNCS *p* ^adjusted^ *=* 0.02; RCS > NR *p* ^adjusted^ = 0.02) (see Figure 2C) and change in arm muscle function (RCS > NR, *p* ^adjusted^ = 0.04) (see Figure 2B).

Notably, the patients classified as RCS according to the functional scales’ thresholds improved not only on the functional scales but also according to their perception.

## 4. Discussion

We found that the global impression of change did not differ between treated patients with SMA classified as responders or non-responders by standardized assessment. Patients’ perceptions of change differed between RCS and NR in only 5 out of 21 questions, with NR reporting an improvement in a broad range of domains. Minimal changes considered as not clinically relevant according to functional scales administered by an HCP might nevertheless be perceived as significant by patients themselves as they understand the progressive nature of their condition [29,30]. It must also be noted that a lack of deterioration can be considered as an achievement in SMA even though the MCID is not reached [27].

Another rationale for considering clinical significance at an individual level, rather than relying on absolute thresholds applicable to the entire population, is that the perception of change depends on the patient’s functional abilities and age at treatment initiation [29,31,32,33].

Our findings support the need to change current appellations related to treatment efficiency. “Non-responders” should not be defined as patients who do not improve by a certain score on a certain scale but as patients for whom the motor, respiratory, and/or bulbar evolution does not diverge from natural history or the expected evolution in the absence of treatment.

The main variations in perception were observed in the upper limb function and trunk balance.

HFMSE was the most used scale, and trunk strength and upper limb function were captured by several items (e.g., sitting items without support, rolling items, four-point kneeling items) in the total score. Patients across the three groups reporting difficulties at the baseline perceived their upper limb function to be improved, which aligns with existing findings highlighted by patients treated with nusinersen [34,35]. Patients assigned to the NR group and who experienced significant improvements in upper limb function could possibly have been identified as responders by the RULM, which is more sensitive to positive upper limb function changes than HFMSE. Indeed, in the pivotal trial of risdiplam, RULM could identify a significant progressive improvement difference in treated patients, while HFMSE failed to achieve this [24].

Our study cohort is broadly heterogeneous regarding the age and types of SMA and composes one of the rare real-world evidence studies, including a pediatric and adult population with all types of SMA [15], which can be perceived as a limitation. Compared to RCS, NRs were older at treatment initiation with a longer disease duration and less access to physiotherapy, which are known poor prognostic factors to treatment response [26]. Beyond the lower effect size of treatments on classical scale changes, treatment efficacy in older patients is always more challenging to demonstrate. This is linked to the irreversible damage of severe and chronic weakness, such as contractures, as well as from a respiratory perspective, comprising recurrent infections and pneumonia [24]. Nevertheless, this study clearly highlights that the absence of measurable responses in patients when using classical outcomes does not rule out significant benefits, as reported by the patients themselves or by their caregivers.

This confirms the need for the use of Patient-Reported Outcome Measures (PROMs) [36] to complement standardized assessments. Several different PROMs and QoL questionnaires have been developed in recent years in SMA [24,36] but also in other neuromuscular conditions [37] to offer a more patient-centric approach. However, the correlation of the QoL questionnaire or PROMs with physical abilities can be challenging, especially in a treated adult population demonstrating only small functional changes on functional scales [38]. Several reasons can explain this poor correlation, such as the fact that clinical assessments are time-point evaluations and PROMs are more integrative measures. In addition, PROMs might be impacted by various variables, such as social and mental well-being [39,40,41].

The additional challenge with studying patient perception throughout a clinical trial is that the results can be biased by initial treatment expectations evolving and changing over time. This could explain why patients not reporting the symptom/difficulty before treatment initiation report a positive change 15 months after treatment initiation. Expectations are related to various factors like type of SMA and functional status [33], but it has also been reported that expectations from patients and caregivers increased over time [30]. In addition, caregivers tend to depreciate the actual well-being of patients [39]. This could explain the fact that patients’ and patients’ caregivers’ overall impression of change in QoL in the RCS group rated no improvement after 15 months of nusinersen, although their functional scales score changed meaningfully. This suggests that the actual thresholds used for meaningfulness are not applicable to all patients. Meaningfulness should be considered at an individual level where the patient and clinician set goals to provide a balance between a patient-centric approach and objectivity.

Our study has several limitations. First, the choice of outcome measures and scales is not universal. We attempted to minimize this limitation using a priori strict algorithm for scale selection. The second limitation is related to the possible heterogenicity in patient ranking. Although the patients were followed up in reference neuromuscular centers, physiotherapists did not undergo study-specific and common training. Time points for assessments are less precise in real life than during clinical trials. The baseline point was collected retrospectively from electronic health records. Furthermore, the list of items on which patients answered through the PGIC did not constitute a validated questionnaire or scale but much more a list of domains, usually listed during clinics. RULM is an outcome measure that is more sensitive to upper limb change and could have captured improvements in the shoulder that were not captured by HFMSE. As HFMSE was prioritized over RULM to classify our patients, this might potentially explain the significant difference between the patient groups. Finally, a longitudinal prospective study would potentially help to better appreciate the evolution in time between perceived and measured efficacy.

## 5. Conclusions

This study demonstrates that the benefit of a treatment, as experienced and reported by patients with SMA and their caregivers, is very poorly related to responder status provided by changes in standardized scale scores. This supports the identification of patient-adapted therapeutic objectives rather than universal thresholds on standardized scales and questions the use of these thresholds as the criteria used by payers to determine treatment continuity.

## Figures and Tables

**Table 1:** Patient characteristics.

	Total (n = 99)	RCS (n = 41)	RNCS (n = 18)	NR (n = 40)	Raw *p*-Value
Age at diagnosis (y);	2.36;	2.36;	1.77;	3.7;	0.47
[Median (min–max)]	(0.01–28.5)	(0.01–28.5)	(0.49–16.5)	(0.23–22.0)	
Age of first symptoms (y);	1.25;	1.42;	1.0;	1.33;	0.29
[Median(min–max)]	(0.04–16.0)	(0.08–12.0)	(0.33–7.0)	(0.04–16.0)	
Age at treatment initiation (y);	11.16;	5.20;	12.3;	18.1;	
[Median(min–max)]	(0.39–57.4)	(0.39–47.8)	(1.41–48.8)	(0.83–57.4)	RCS < NR, *p* ^adjusted^ ≤ 0.001
Sex (% male)	51.5%	48.8%	50.0%	55.0%	0.85
SMN2 copy, n					
2 SMN2 copies	9.0	5.0	2.0	2.0	0.13
3 SMN2 copies	62.0	31.0	10.0	21.0	
4 SMN2 copies	20.0	3.0	6.0	11.0	
>4 SMN2 copies	3.0	1.0	0.0	2.0	
Unknown	5.0	1.0	0.0	4.0	
Patient type, n					
SMA Type 1 from 3 to 24 months	6.0	4.0	0.0	2.0	
SMA Type 1 from 24 months	5.0	0.0	3.0	2.0	0.17
SMA Type 2 from 6 to 24 months	2.0	1.0	0.0	1.0	
SMA Type 2 from 24 months	32.0	11.0	8.0	13.0	
SMA Type 3 from 24 months	11.0	7.0	0.0	4.0	
SMA Type 3 from 36 months	44.0	18.0	7.0	18.0	
Ambulatory status, n					
Non-ambulant	58.0	20.0	13.0	25.0	0.30
Loss of ambulation in progress	9.0	6.0	0.0	3.0	
Ambulant	32.0	15.0	5.0	12.0	
AE, n					
Hernia/fractures	12.0%	43.0%	0%	0%	
Spinal surgery ^a^	52.0%	29.0%	88.0%	40.0%	0.06
Hospitalization in ICU	12.0%	14.0%	12.0%	10.0%	
NIV implementation	8.0%	14.0%	0%	10.0%	
Two AEs	4.0%	0.0%	0.0%	10.0%	
Other	12.0%	0.0%	0.0%	30.0%	
Physiotherapy (% with physiotherapy)	78.8%	92.7%	61.1%	72.5%	RCS > NR, RNCS < NR, *p* ^adjusted^ = 0.01
Contractures (% with contracture)					
Left wrist/hand	15.2%	2.4%	27.8%	22.5%	RCS < RNCS, *p* ^adjusted^ = 0.01
Right wrist/hand	15.2%	0.0%	33.3%	22.5%	RNCS > NR, *p* ^adjusted^ = 0.001
Left elbow	23.2%	14.6%	33.3%	27.5%	0.21
Right elbow	23.2%	14.6%	33.3%	27.5%	0.21
Left shoulder	10.1%	2.4%	22.2%	12.5%	0.06
Right shoulder	10.1%	2.4%	22.2%	12.5%	0.06
Left hip	41.4%	31.7%	61.1%	42.5%	0.11
Right hip	41.4%	31.7%	61.1%	42.5%	0.11
Left knee	52.5%	51.2%	50.0%	55.0%	0.92
Right knee	51.5%	51.2%	44.4%	55.0%	0.76
Left ankle	48.5%	36.6%	55.6%	57.5%	0.14
Right ankle	44.4%	31.7%	50.0%	55.0%	0.09
Neck	10.1%	0.0%	22.2%	15.0%	0.13
Scoliosis (% with scoliosis)	64.6%	56.1%	83.3%	65.0%	0.13
All spinal surgery ^b^ (% with spinal surgery)	27.3%	9.8%	38.9%	40.0%	RCS < RNCS, RCS < NR, *p* ^adjusted^ = 0.03
Ventilatory assistance (% with assistance)	23.2%	14.6%	44.4%	22.5%	RCS < RNCS, RNCS > NR, *p* ^adjusted^ = 0.04
% with diurnal assistance	23.0%	17.0%	37.0%	12.5%	0.45
% with nocturnal assistance	87.0%	100.0%	87.0%	78.0%	0.45
Feeding assistance (% with assistance)	7.10%	4.90%	5.60%	10.0%	0.64

Participant characteristics. RCS = responder clinically significant, RNCS = responder non-clinically significant, and NR = non-responder. ^a^ Spinal surgery after treatment initiation. ^b^ All spinal surgeries before and after treatment initiation. Significant values that survived Bonferroni correction for multiple comparisons are in bold. Adjusted significance with *p*-value < 0.05.

**Table 2:** Difference between baseline and M15 on the prioritized scale in relation to age at treatment initiation and patient group.

Assessment Type	Age Category	RCS (n = 41)	RNCS (n = 18)	NR (n = 40)
CHOP-INTEND (Δ)	Children (<18 y)	16 ± 4.74 (n = 5)	2.0 1.0 (n = 3)	−2.0 ± 2.0 (n = 3)
	Adult (≥18 y)	–	–	–
HINE-2 (Δ)	Children (<18 y)	–	–	−0.50 ± 0.71 (n = 2)
	Adult (≥18 y)	–	–	–
HFMSE (Δ)	Children (<18 y)	8.4 ± 4.5 (n = 22)	1.6 ± 0.52 (n = 10)	−1.82 ± 2.14 (n = 11)
	Adult (≥18 y)	7.6 ± 5.4 (n = 5)	2.0 (n = 1)	−1.54 ± 1.94 (n = 13)
MFM32 (Δ)	Children (<18 y)	–	2.0 (n = 1)	−6.0 (n = 1)
	Adult (≥18 y)	–	–	–
MFM20 (Δ)	Children (<18 y)	8.67 ± 3.2 (n = 3)	–	–
	Adult (≥18 y)	–	–	–
RULM (Δ)	Children (<18 y)	5.0 ± 2.8 (n = 2)	1.0 ± 0.0 (n = 2)	−1.0 ± 1.4 (n = 2)
	Adult (≥18 y)	3.0 ± 1.4 (n = 2)	1.0 ± 0.0 (n = 1)	−1.0 ± 1.0 (n = 5)
6MWT (Δ)	Children (<18 y)	91.0 (n = 1)	–	−8.0 (n = 1)
	Adult (≥18 y)	86.0 (n = 1)	–	−13.0 ± 18.4 (n = 2)

Age category is determined by age at treatment initiation. Functional assessments used to assess the patients: Children’s Hospital of Philadelphia Infant Test of Neuromuscular Disorders (CHOP-INTEND), Hammersmith Infant Neuromuscular Examination-Section 2 (HINE-2), Hammersmith Functional Motor Scale—Expanded (HFMSE), Revised Upper Limb Module (RULM), Motor Function Measure (MFM32), 6 min walk test (6MWT). Delta (Δ) of baseline and month 15. Score changes (Δ) after 15 months of treatment initiation depending on age category at treatment initiation across the patient groups are presented.

**Table 3:** Perception of patients or caregivers for each of the 21 questions of the questionnaire across the responder groups depending on the presence of symptoms/difficulty before treatment initiation.

Questions (n = 21)	Symptom/Difficulty before Treatment Initiation? (Baseline Question)	RCS (n = 41)	RNCS (n = 18)	NR (n = 40)	Raw *p*-Value
		Mean Rank			
Change in tremor	Yes	33.8 (n = 32)	40.5 (n = 12)	29.3 (n = 22)	0.23
	No	– (n = 9)	– (n = 6)	– (n = 18)	
Change in balance while sitting	YES	26.2 (n = 18)	19.8 (n = 9)	18.5 (n = 16)	0.16
	No	32.5 (n = 23)	28.7 (n = 9)	24.5 (n = 24)	RCS > NR, *p* ^adjusted^ = 0.04
Change in balance while standing/walking	Yes	43.2 (n = 34)	35.1 (n = 14)	35.9 (n = 29)	0.32
	No	– (n = 7)	– (n = 4)	– (n = 11)	
Change in fatigue	Yes	22.0 (n = 19)	26.2 (n = 9)	17.8 (n = 14)	0.22
	No	28.6 (n = 22)	31.7 (n = 9)	28.4 (n = 26)	0.76
Change in function involving hand and wrist muscles	Yes	32.7 (n = 23)	29.9 (n = 11)	23.4 (n = 22)	0.13
	No	24.6 (n = 18)	23.2 (n = 7)	18.9 (n = 18)	0.07
Change in function involving arm muscles	Yes	35.1 (n = 22)	21.9 (n = 9)	23.7 (n = 24)	RCS > NR, *p* ^adjusted^ = 0.04
	No	24.4 (n = 19)	23.4 (n = 9)	19.7 (n = 16)	0.14
Change in function involving shoulder muscles	Yes	42.7 (n = 26)	26.4 (n = 13)	29.4 (n = 28)	RCS > RNCS, *p* ^adjusted^ = 0.02 RCS > NR, *p* ^adjusted^ = 0.02
	No	18.9 (n = 15)	18.9 (n = 5)	12.5 (n = 12)	RCS > NR, *p* ^adjusted^ = 0.04
Change in ability to eat	Yes	17.7 (n = 12)	18.3 (n = 7)	15.7 (n = 14)	0.79
	No	33.5 (n = 29)	33.5 (n = 11)	33.5 (n = 26)	1.00
Change in appetite	Yes	– (n = 9)	– (n = 6)	– (n = 10)	
	No	40.1 (n = 32)	38.1 (n = 12)	34.5 (n = 30)	0.24
Change in aspiration	Yes	– (n = 10)	– (n = 5)	– (n = 6)	
	No	40.7 (n = 31)	39.5 (n = 13)	38.4 (n = 34)	0.55
Change in swallowing	Yes	– (n = 9)	– (n = 4)	– (n = 11)	
	No	37.5 (n = 32)	37.5 (n = 14)	38.8 (n = 29)	0.76
Change in chewing	Yes	15.5 (n = 11)	14.6 (n = 6)	12.1 (n = 10)	0.59
	No	36.0 (n = 30)	36.0 (n = 12)	37.2 (n = 30)	0.87
Change in loudness of voice	Yes	– (n = 8)	– (n = 6)	– (n = 8)	
	No	42.1 (n = 33)	37.5 (n = 12)	36.4 (n = 32)	RCS > NR, *p* ^adjusted^ = 0.04
Change in capacity for continuous conversation	Yes	– (n = 8)	– (n = 6)	– (n = 5)	
	No	42.6 (n = 33)	39.0 (n = 12)	39.0 (n = 35)	0.26
Change in quality of sleep	Yes	18.5 (n = 19)	22.3 (n = 6)	14.6 (n = 10)	0.24
	No	33.4 (n = 22)	32.0 (n = 12)	32.0 (n = 30)	0.82
Change in need for nocturnal ventilation	Yes	– (n = 6)	– (n = 6)	– (n = 9)	
	No	40.1 (n = 35)	42.3 (n = 12)	37.8 (n = 31)	0.35
Change in diurnal ventilation	Yes	– (n = 2)	– (n = 3)	– (n = 4)	
	No	45.9 (n = 39)	46.0 (n = 15)	44.8 (n = 36)	0.78
Change in cough	Yes	23.0 (n = 15)	21.6 (n = 8)	17.7 (n = 17)	0.39
	No	29.5 (n = 26)	29.5 (n = 10)	30.8 (n = 23)	0.77
Change in frequency of respiratory infections	Yes	18.9 (n = 13)	19.2 (n = 8)	18.9 (n = 16)	0.99
	No	30.8 (n = 28)	33.0 (n = 10)	31.7 (n = 24)	0.78
Change in the frequency of hospitalization due to respiratory infection	Yes	– (n = 7)	– (n = 5)	– (n = 10)	
	No	39.0 (n = 34)	39.0 (n = 13)	39.0 (n = 30)	1.00
Change in recurrent disease-related pain	Yes	17.7 (n = 16)	18.8 (n = 3)	13.1 (n = 12)	0.33
	No	34.0 (n = 25)	34.0 (n = 15)	35.2 (n = 28)	0.93

The number of patients or caregivers who reported (yes or no) noticing symptoms or difficulty and changes prior to treatment initiation divided across 3 patient groups. Question 1 is excluded from this analysis; 21 questions remained. Statistical analysis was performed only when each questionnaire item (reporting yes or no) had a minimum of 10 patients represented across at least two patient groups. The perception of patients was scored using the Patient Global Impression of Change (PGIC) presented as the mean rank and as examined by the Kruskal–Wallis test. Significant results that survived the Bonferroni correction for multiple comparisons are in bold. Adjusted significance with *p*-value < 0.05.

## Data Availability

The original contributions presented in this study are included in the article/Supplementary Materials; further inquiries can be directed to the corresponding authors.

## Supplementary Materials

The following supporting information can be downloaded at https://www.mdpi.com/article/10.3390/jcm13123418/s1, Figure S1: Main results analysis flowchart; Table S1: Inclusion criteria: study windows and functional scales; Table S2: Assignment to responder groups; Table S3: Questionnaire; Table S4: Perception of patients for each question of the questionnaire across the responder groups.
