# Supplementary material for: Patients’ Perceptions of Nusinersen Effects According to Their Responder Status
Source: J Clin Med. 2024 Jun 11;13(12):3418. doi: 10.3390/jcm13123418 (PMC11205004; doi:10.3390/jcm13123418)
Supplement: Supplementary file 1 [file jcm-13-03418-s001.zip › jcm-3027026-supplementary.pdf]

Supplementary Materials

Figure S1: Main results Analysis Flowchart

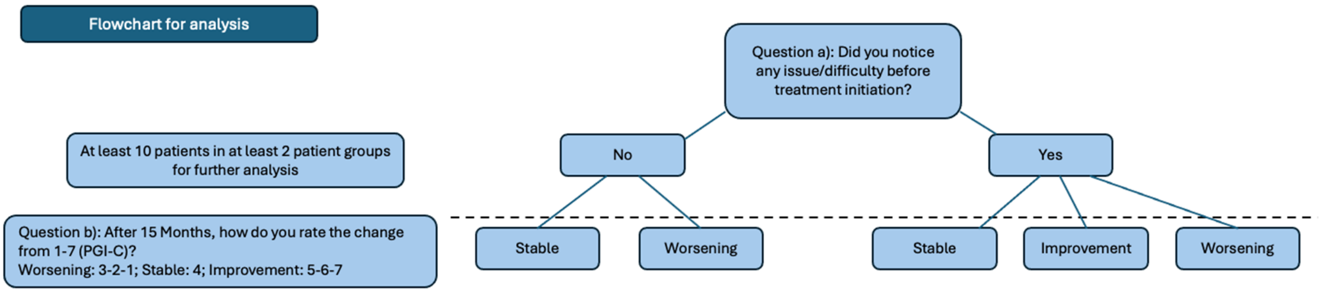

Presence or not of symptom/difficulty before treatment initiation helps to determine the patient's trajectory depending on perception.

Table S1: Inclusion criteria: Study windows and Functional Scales

**Study windows**		
Baseline		A baseline visit is accepted when performed within three months before treatment initiation. If no assessment before treatment initiation exists, an assessment is accepted as baseline if performed within two weeks after treatment is initiated with the first Spinraza injection.
Month 15		A Month 15 visit is accepted when performed either on the fifteenth month after treatment initiation, or if no assessment at Month 15 is possible, the next closest assessment thereafter.
**The order of priority of scales according to age at treatment initiation and type of SMA**		
Age at treatment initiation	SMA type	Scales in order of priority
From 3 – 24 months	Type I ('non-sitter')	1. CHOP-INTEND<br>2. HINE-2
From 6 – 24 months	Type II ('sitter')	1. CHOP-INTEND<br>2. HINE-2
From 18 – 24 months	Type III ('walker')	1. CHOP-INTEND<br>2. HINE-2
From 24 months	Type I	1. CHOP-INTEND<br>2. HINE-2<br>3. HFMSE<br>4. MFM32/MFM20<br>5. RULM
From 24 months	Type II	1. HFMSE<br>2. MFM32/MFM20<br>3. RULM

From 24 months	Type III	1. HFMSE<br>2. MFM32/MFM20<br>3. RULM
From 36 months	Type III	1. HFMSE<br>2. 6MWT<br>3. MFM32/MFM20<br>4. RULM

List of validated functional scales: Children's Hospital of Philadelphia Infant Test of Neuromuscular Disorders (CHOP-INTEND); Hammersmith Infant Neurological Examination – Section 2 (HINE-2); Hammersmith Functional Motor Scale Expanded (HFMSE); Motor Function Measure 32 and 20 (MFM32; MFM20; Revised Upper Limb Module (RULM); 6-minute walk test (6MWT).

All scales have been used in SMA-related clinical trials for disease-modifying therapies and are usually recommended in clinical follow-up of patients treated with Spinraza. CHOP-INTEND was considered and accepted for all non-sitter patients regardless of their age because it is still commonly used in clinical follow-up for older non-sitter patients. The 6MWT has been widely used to assess functional exercise capacity in ambulatory patients with SMA. However, to be consistent with the other categories, motor function scales like the HFMSE instead of the 6MWT for the ambulant patients were prioritized.

Table S2: Assignment to Responder groups

Scales	Month 15: point change to be considered as Responder clinically significant (RCS)	Month 15: point change to be considered as Responder non-clinically significant	Month 15: point change to be considered as Non-responder (NR)
CHOP-intend	>= 4 points	< 4 points; > 0 points	0 points/meters; loss of points/meters
HINE-2	>= 2 points	< 2 points; > 0 points	
HFMSE	>= 3 points	< 3 points; > 0 points	
MFM32	>= 3 points	< 3 points; > 0 points	
RULM	>= 2 points	< 2 points; > 0 points	
6MWT	>= 30 meters	< 30 meters; > 0 meters	

The scores thresholds considered as clinically significant improvement according to each functional scale based on literature and our classification.

Table S3: Questionnaire

Question	Domain	Question	Domain	Question	Domain
1	General Impression	9	Ability to eat a whole meal	17	Nocturnal ventilation support
2	Tremor	10	Appetite	18	Diurnal ventilation support
3	Balance while sitting	11	Frequency of aspiration during the day	19	Coughing
4	Balance while standing/waking	12	Swallowing	20	Frequency of respiratory infections
5	Fatigability	13	Chewing	21	Frequency of hospitalizations due to respiratory infections
6	Functions that involve the muscles of hands and wrists (writing, typing, using a joystick, drawing, colouring, etc.)	14	Loudness of voice	22	Pain
7	Functions that involve the muscles of arms (eating alone, brushing teeth, combing hair, applying make-up, etc.)	15	Having a continuous conversation		

8	Functions that involve the muscles of shoulders (washing the hair, reaching objects above the head, etc.)	16	Quality of sleep

Questionnaire: Each question refers to one specific aspect/domain of patient's quality of life and is scored with the PGIC scoring system.

Table S4: Perception of patients for each question of the questionnaire across the responder groups

Questions (n=22)	RCS (n= 41)	RNCS (n=18)	NR (n= 40)	Raw p-value
	Mean Rank			
Change in quality of life	46.9	54.9	46.9	0.57
Change in tremor	56.3	50.6	43.3	0.07
Change in balance while sitting	57.1	51.7	41.9	**RCS > NR $p^{adjusted}$=0.03**
Change in balance while standing/walking	56.7	43.7	45.9	0.12
Change in fatigue	50.6	60.0	44.9	0.14
Change in function involving hand and wrist muscles	55.3	54.1	42.7	0.08
Change in function involving arm muscles	58.2	43.4	44.6	0.03, *RCS>NR $p^{adjusted}$ =0.06
Change in function involving shoulder muscles	59.90	44.03	42.54	**RCS>NR, $p^{adjusted}$=0.006**
Change in ability to eat	50.7	52.2	48.3	0.79
Change in appetite	50.5	55.8	46.9	0.39
Change in aspiration	55.6	50.8	43.9	**RCS>NR, $p^{adjusted}$=0.024**
Change in swallowing	50.4	50.7	49.2	0.95
Change in chewing	51.6	52.4	47.3	0.58
Change in loudness of voice	52.3	55.3	45.2	0.19
Change in capacity for continuous conversation	51.5	58.7	44.5	0.06
Change in quality of sleep	57.0	50.6	42.5	**RCS > NR, $p^{adjusted}$=0.01**
Change in need for nocturnal ventilation	51.8	52.5	47.0	0.26
Change in diurnal ventilation	49.7	56.6	47.3	**RNCS > NR, $p^{adjusted}$=0.03**
Change in cough	50.8	51.8	48.4	0.86
Change in frequency of respiratory infections	47.8	51.7	51.5	0.76
Change in the frequency of hospitalization due to respiratory infection	48.1	54.8	49.9	0.53
Change in recurrent disease-related pain	53.1	47.9	47.8	0.55

Perception of patients scored by using the Patient Global Impression of change (PGIC) presented in mean rank by using Kruskal Wallis test. Significant results had a Bonferroni correction for multiple comparisons and are in bold. Significances with adjusted p-value <0.05 are in bold. *Adjusted p-value for change involving the arm muscles is not significant after Bonferroni correction.
